# Predictors of Readmission in Young Adults with First-Episode Psychosis: A Multicentric Retrospective Study with a 12-Month Follow-Up

**DOI:** 10.3390/clinpract14040099

**Published:** 2024-06-24

**Authors:** Filippo Besana, Serena Chiara Civardi, Filippo Mazzoni, Giovanni Carnevale Miacca, Vincenzo Arienti, Matteo Rocchetti, Pierluigi Politi, Vassilis Martiadis, Natascia Brondino, Miriam Olivola

**Affiliations:** 1 Department of Brain and Behavioural Sciences, University of Pavia, 27100 Pavia, Italy; serenachiara.civardi01@universitadipavia.it (S.C.C.); filippo.mazzoni01@universitadipavia.it (F.M.); giovanni.carnevalemiacc01@universitadipavia.it (G.C.M.); vincenzo.arienti01@universitadipavia.it (V.A.); pierluigi.politi@unipv.it (P.P.); natascia.brondino@unipv.it (N.B.); miriamolivola@icloud.com (M.O.); 2 Department of Mental Health and Addiction, ASST Pavia, 27100 Pavia, Italy; matteo_rocchetti@asst-pavia.it; 3 Department of Mental Health, ASL Napoli 1 Centro, 80145 Naples, Italy; vassilis.martiadis@gmail.com

**Keywords:** first-episode psychosis, long-acting antipsychotics, readmission, youth mental health

## Abstract

Background: A significant number of young individuals are readmitted one or more times shortly after their first episode of psychosis. Readmission may represent a marker of psychopathological vulnerability. Our primary aim was to evaluate the impact of clinical and socio-demographic variables on readmission at 12-month follow-up. Secondly, our goal was to determine whether the use of Long-Acting Injection (LAI) antipsychotics provides notable benefits compared to oral medications in preventing subsequent readmissions. Subjects and methods: 80 patients hospitalised for the first time with a diagnosis of psychotic disorder (ICD-10 criteria) were retrospectively assessed through clinical records. The mean age was 21.7 years. Patients were predominantly male (n = 62, 77.5%), and 55 subjects had at least 8 years of education. 50% of the sample was “NEET” (not in education, employment, or training). Results: 35 patients (43.8%) were discharged with a LAI antipsychotic, while 45 (56.2%) recieved oral antipsychotic therapy. Substance use (*p* = 0.04) and oral antipsychotics at discharge (*p* = 0.003) were significantly associated with readmission at 1 year. We did not find any significant predictors of being discharged with LAI therapy. Conclusion: Our findings underlined the importance of identifying patients at risk of readmission in order to prevent future rehospitalization and promote appropriate prevention strategies. LAIs should be considered as a first-choice treatment for patients hospitalised for FEP since they proved to be effective in preventing relapse.

## 1. Introduction

The periods of late adolescence and early adulthood are of significant importance from a psychological standpoint, as they signify the onset of numerous mental disorders [1]. In this context, psychotic disorders stand out as a notable area of focus, given their tendency to initiate during the stages of adolescence and early adulthood [2]. In order to prevent, or at least reduce, the consequences of being diagnosed with a psychotic disorder, it is important to detect people at high risk for these conditions [3,4,5]. Several evidence-based programmes have been developed to prevent situations at risk of psychosis. Specifically, it is worth mentioning the OASIS programme in London (UK) [6] and the ORYGEN programme in Melbourne, Australia [7]. In Italy, one of the most important psychosis prevention services was the ‘Parma At-Risk Mental States’ (PARMS) programme [8].

However, if the first psychotic episode (FEP) has already taken place, early intervention strategies must be set up to reduce the duration of untreated psychosis (DUP) [9], that is, the time from the first outburst of psychotic symptoms to the initiation of appropriate treatment with antipsychotic medications [10]. It has been postulated that a delay in initiating treatment is associated with a poorer response and a worse outcome [11]. Treatment delay can be attributed to various factors, such as the fear of stigma. cultural beliefs, familiar attitudes, and a lack of education [12]. Hospitalisation is frequently used as a study outcome, and some authors have found that a large portion of patients require readmission after FEP [13,14]. These readmissions are quite common during the first years following a psychotic outbreak [15], and rehospitalization should thus be regarded as a proxy for treatment failure [16]. This holds particular relevance for youthful patients, as experiencing relapses within the initial years following a psychotic episode serves as an indicator of future clinical and functional outcomes in the long term [17].

Recent data on readmission rates among youths showed that more than 30% of young patients are readmitted to acute psychiatric inpatient units within one year after discharge, regardless of their diagnosis of admission [18]. Regarding psychotic disorders, early readmission can be considered an indicator of psychopathological vulnerability [19]. A recent meta-analysis focusing on psychiatric readmission of children and adolescents identified suicidal ideation at first admission, prior hospitalization, and discharge from a residential treatment as factors associated with relapse [20].

Of note, a recent study [21] found that 67% of FEP patients experience readmission in the first year. It is crucial to shed more light on variables potentially impacting the readmission rate in this population. For instance, treatment compliance is generally considered a determinant prognostic factor. In this context, Long-Acting Injection (LAI) may represent a first-choice treatment, as a lot of patients with psychosis tend to show poor adherence to treatment [22]. Researchers have analysed the role of LAI therapy and its potential advantages over oral medications, even in patients with poor prognosis [23,24,25,26]. In this group of patients, LAI antipsychotics provided a relapse rate similar to that of those patients with a better prognosis who were treated with oral medications. Another potential predictor is the presence of substance use in people with FEP. This phenomenon is quite common and has significant implications in terms of psychopathology, namely positive symptoms [27,28] and clinical outcomes [29,30]. Substance abuse has been associated with higher relapse and readmission rates [31,32]. Specifically, drug abuse causes impairment in cortical and subcortical regions that are responsible for decision-making and risk taking behaviour, such as the ventro-medial prefrontal cortex, amygdala, and caudate nucleus. These neurobiological dysfunctions contribute significantly to relapses and new hospitalisations, with the associated direct and indirect costs [33,34].

On the whole, evidence has shown that this pattern is reversible, with patients achieving abstinence [35]. In particular, the study by Weibell and colleagues [36] showed that patients who became abstinent during the first two years after a psychotic outbreak had similar outcomes compared to subjects who had never used substances. Moreover, substance use and adherence to therapy are closely linked [37]. Previous research also identified younger age [38], unmarried status [37,39], unemployment [37,40] and longer admission days [39] as factors associated with 1-year readmission. A Japanese study highlighted that a higher number of hospitalisations and higher scores on the Schizophrenia Cognition Rating Scale Japanese version (SCoRS-J), assessing cognitive functions in schizophrenia, were identified as risk factors for readmission [41]. Furthermore, it was highlighted that the risk of readmission in patients with severe mental disorders was increased by 25% for those treated with typical antipsychotics, compared to those who used atypical ones [42].

While several studies focused on the predictors of readmission in the general population, less research is focused on younger age, and specifically, on young people admitted for a First Episode-Psychosis. There is a need for more research on this group of patients in order to implement personalised relapse prevention strategies and tailored treatment programmes [43].

With this in mind, we performed a study aimed at evaluating specific risk factors for early readmission in FEP patients, focusing on young adults aged 18–25 years. This age group deserves special attention, as it can be the ideal target for early intervention strategies.

## 2. Materials and Methods

### 2.1. Study Population, Setting and Design

Our study is observational and multicentric, with a retrospective design and a 12-month follow-up. Data were retrospectively collected from interviews and clinical records of patients admitted to three psychiatric inpatient units. These units belong to the Department of Mental Health and Addiction of ASST Pavia, Pavia, Italy, between 1 January 2016 and 31 December 2021 (covering a catchment area of 535,493 inhabitants) and are located in three different towns: Pavia, Voghera, and Vigevano. There are no specific age criteria for admission, although generally a minimum age limit for admission is considered to be 16 years. When patients are discharged from the hospital, they are referred to the local psychiatric outpatient clinics, where a multidisciplinary care pathway is implemented with psychiatric, psychological, and social assessments. A specific micro-equipe for each client is assigned based on a case management model. Generally, a micro-equipe is composed of a psychiatrist, a psychologist, a social worker, and a professional nurse. Especially for young people, even a psychiatric rehabilitation therapist is assigned in order to achieve a more tailored recovery-oriented approach. In the local psychiatric outpatient clinics, it is also possible to administer the LAI therapy at a predetermined frequency. Furthermore, some rehabilitative and socialising activities are implemented, such as psycho-education groups for patients with bipolar disorder and schizophrenia, social skills training, and reading or discussion groups. The decision to take part in a group activity is agreed upon with the patient based on his or her personal characteristics and aptitudes. When useful and appropriate, with the patient’s consent, the patient’s family or caregivers are also involved in family meetings or group activities. If necessary, especially in order to promote compliance in young people, supervision in drug therapy assumption in the first weeks after hospitalisation is arranged daily at the mental health centre, coordinated by the nursing staff, as well as a daily interview with the client, in order to create a successful therapeutic alliance.

Socio-demographic and psychopathological data were collected from clinical records and were cross-checked by at least two authors in order to avoid collection mistakes (FM, GCM, VA). If no consensus was reached, a third member of the research team was consulted.

The inclusion criteria were: (1) patients hospitalized for a First-Episode Psychosis (FEP); (2) aged between 18 and 25 years old at the time of hospitalisation. Subjects with missing or incomplete clinical records were excluded (N = 25).

The diagnosis was formulated by the attending psychiatrists following the ICD10 criteria. Included conditions were the following: F19.5 (psychotic disorders due to psychoactive substance use), F21 (schizotypal disorder), F23 (acute and transient psychotic disorders), F28 (other nonorganic psychotic disorders), F29 (unspecified nonorganic psychosis), F30.2 (manic episodes with psychotic symptoms) and F32.3 (severe depressive episodes with psychotic symptoms). Our investigation was conducted in accordance with the ethical principles of the Declaration of Helsinki, and the protocol of the study was approved by the local ethical committee.

In accordance with previous research [37,44,45], we defined readmission as being readmitted into the hospital within 12 months after the first hospitalisation for FEP. Readmission rates were evaluated at two different time points: 6 months (T1) and 12 months (T2) from baseline (T0). Eventually, 80 subjects (62 M and 18 F) were included in the study.

The socio-demographic characteristics of the sample are summarised in Table 1. The mean age of the sample was 21.7 years. Most patients were male (n = 62, 77.5%), and 55 subjects had at least 8 years of education (secondary school). 50% of the sample was registered as NEET (not in education, employment, or training). 88.7% (N = 71) were not in a relationship at the time of admission.

### 2.2. Assessment and Clinical Evaluation

Psychopathology was evaluated by means of the Positive and Negative Syndrome Scale [46] both on admission and discharge. This 30-item scale consists of an interview to assess the symptoms and severity of patients affected by schizophrenia and other psychotic disorders. Each item ranges from 1 to 7 (1 = absent, 2 = minimal, 3 = mild, 4 = moderate, 5 = moderate-severe, 6 = severe, and 7 = extreme).

The following anamnestic and clinical information was collected during interviews: marital status; education; occupational or academic status; family and/or personal history of psychiatric disorders; previous evaluations carried out by psychiatrists and/or psychologists; time of onset and duration of the current psychiatric symptoms; presence of potential medical comorbidities and/or allergies, with particular reference to drug allergies; comorbid alcohol or substance use disorder; legal issues before admission (we considered a “legal issue” a matter with criminal implications). Robbery and physical assault were the major legal problems in which our selected patients were implicated. All data were collected in an anonymised form.

### 2.3. Statistical Analysis

First, a descriptive analysis of the selected variables was carried out. Subsequently, Wald’s conditional logistic regression models were constructed using readmission within one year as the dependent variable and all the sociodemographic variables, PANSS score at admission, and type of discharge therapy (oral vs. LAI) as the independent variables. Another Wald’s conditional logistic regression was conducted to determine predictors of therapy with LAI at discharge. A two-tailed *p*-value < 0.05 was regarded as statistically significant. The data were analysed using the Jamovi program, version 2.3 [47].

## 3. Results

### 3.1. Clinical Characteristics

Psychopathological features are reported in Table 2. The most frequent diagnosis was schizophrenia spectrum disorder (n = 52, 65%). 24 subjects (25%) had a comorbid substance use disorder, while 14 patients (17.5%) had a family history of mental disorders. Only 38 patients had previous contact with mental health services prior to hospitalisation. Five subjects had legal problems before the admission. 35 (43.8%) were discharged with LAI therapy, while 45 (56.2%) recieved an oral antipsychotic. In the 12-month period of follow-up, no patient in our study committed suicide, and no admission to prison for crimes was detected.

We observed 19 (23.8%) readmissions at 6 months, 36 (21.3%) at 1 year. Five patients were readmitted two times (Table 3).

### 3.2. Logistic Regression Model

Wald’s conditional logistic regression using readmission within one year as the dependent variables yielded a significant model (Chi-square = 13.93, *p* = 0.01, Likeness-log = 92.81, R^2^ = 0.217), which explained 22% of the variance of the dependent variables. The significant independent predictors were lifetime substance use disorder (OR = 2.92; 95% CI: 1.04; 8.18; *p* = 0.04) and being discharged with an oral antipsychotic (OR = 4.95; 95% CI: 1.72; 14.22; *p* = 0.003). To examine the factors linked to being discharged with a LAI, we constructed a logistic regression model in which the type of therapy at discharge (LAI or oral) was used as the dependent variable (Table 4). We did not obtain any significant models.

## 4. Discussion

Our study assessed how various predictors impact readmission following a first-episode psychosis (FEP) at the 1-year follow-up. In our study, 45% of the sample (36 subjects) was readmitted within 1 year after FEP. This datum is slightly higher than that reported by Robinson and colleagues, who reported a 2-year readmission rate between 34 and 37% [48]. However, on the other hand, our finding is lower than another study [21], which showed a 1-year readmission rate of 67% in patients with FEP.

We observed a positive association between readmission and substance abuse disorder in patients aged 18–25. This result is in line with previous literature on the topic, which focused on the role of cannabis use in predicting readmission [1,48,49,50]. Specifically, we chose to focus on younger patients in order to highlight the importance of integrated and tailored treatment plans for a successful relapse prevention strategy in this category of patients. Clinicians should be particularly careful in investigating cannabis and synthetic cannabinoids abuse when treating young patients at their first psychotic episode, given their common use in the youth population and their clear association with psychotic relapse [51]. These findings underline the importance of implementing primary and secondary prevention strategies regarding psychoactive substance use, as well as promoting cooperation between general psychiatry and addiction mental health services [52,53]. In this light, it would be interesting in future research to compare the readmission rates in countries where the status of legislation concerning drug use is different.

Interestingly, we found a protective effect of LAI therapy on readmission rates. LAIs are frequently introduced when oral therapy proves ineffective, and they are employed in cases of poor patient adherence, but they are frequently perceived as stigmatising and linked to side effects by clinicians [54,55]. Similarly, it is not easy to introduce LAI therapy in cases where the diagnosis is not certain, and this contributes to the underuse of LAIs [56]. Our finding supports the importance of LAI as a first treatment option, as it may prevent relapse and improve long-term outcomes and compliance [26,57,58]. Similarly, Abdel-Baki and colleagues conducted a longitudinal, 3-year prospective and retrospective study on 237 patients with FEP and substance use disorder treated with oral antipsychotics, or LAI. Although the LAI-AP group presented worse prognostic factors (e.g., history of homelessness), they showed a lower relapse rate, higher relapse-free time, and trends for reduced rehospitalization rates (48.4% vs. 57.3%) [26]. In accordance with these results, one study detected lower odds of readmission in patients with LAI compared to oral medications [59].

We did not find significant predictors for the introduction of LAI versus oral treatment. This finding could be at least in part due to the limited sample size. Additionally, the rate of LAI in our study is moderate, and this could have impacted the results.

Of note, however, the choice of LAI antipsychotics in our sample (43.8%) is more common than in previous research [60]; this could indicate a shifting perception among clinicians and patients regarding the use of LAI therapy as a second-line option, primarily for the most severely affected patients. This notion is further substantiated by the 1-year rehospitalization rate observed in our sample for patients receiving LAI (7 out of 35, 20%), which is lower than the rates reported in earlier literature [26,61].

Furthermore, from a clinical perspective, the selection of LAI antipsychotics should also be guided by considerations of long-term recovery. Indeed, long-acting treatments can enhance adherence and ensure consistent care. These factors hold great significance in advancing long-term objectives, such as enhancing psychosocial functioning, which constitutes a facet of full recovery [62]. This topic has also been addressed in a recent Delphi panel project used to obtain expert consensus [63]. The 17 Delphi panellists, selected among practicing psychiatrists with a special expertise in treating schizophrenia with long-acting therapies, supported the use of LAI antipsychotics in FEP patients. The panel also emphasised their importance in reducing rehospitalization and relapse rates. These benefits are strongly related to longer-term functional recovery.

Our findings should be interpreted cautiously, considering some methodological limitations. Firstly, the limited sample size did not allow us to analyse the pharmacological therapy in more details, e.g., for example, comparing different drugs in different formulations (long-acting or oral), as in other works [64,65], or investigating the effect of specific psychoactive substances. Secondly, our study has adopted a 1-year follow-up: a longer follow-up time could reveal different predictive factors in readmission, also contributing to identifying the so-called “psychiatric revolving door” associated factors, focusing on people readmitted several times in the same period [66,67]. Lastly, it is important to note that the Italian mental health care system relies on a community-based model [68]. Therefore, our results, grounded in a specific geographical catchment area, might have been influenced by unexplored socio-demographic factors that may have impacted the rates of readmission [69]. More comprehensive data from different local areas is needed to provide a nation-wide picture. Similarly, the inclusion of different psychosocial factors in our data, such as family violence, homelessness, and social isolation, could provide a more comprehensive overview of our results.

## 5. Conclusions

The results of the present study could help clinicians develop more personalised interventions to prevent readmission and improve psychiatric care for young people with a psychotic disorder. For instance, psychosocial interventions focused on substance abuse and integrated prevention policies and strategies between different mental health services could offer significant advantages in preventing new readmissions. LAIs, integrated with personalised psychosocial treatment interventions, should be considered as a first-choice treatment for patients hospitalised for FEP since they proved to be effective in preventing relapses, both in the adult and adolescent populations. Further studies, using both qualitative and quantitative design, are needed to identify risk factors for readmission in order to implement appropriate prevention strategies, especially for the young population.

## Figures and Tables

**Table 1:** Socio-demographic characteristics of the sample.

Socio-Demographic Features	
Age (at first hospitalisation)	Mean 21.7 (SD 2.27), range 18–26
Gender	18 F (22.5%), 62 M (77.5%)
Marital status	9 (11.3%) Married or in a relationship
	71 (88.7%) Single
Education	16 (20.0%) less than 8 years
	64 (80.0%) 8 years or more
Occupational status	Not in education, employment, or training 40 (50%)
	Student 18 (22.5%)
	Employed 22 (27.5%)
Legal problems before admission (robbery, physical assault)	Yes 5 (6.2%)
	No 75 (93.8%)

**Table 2:** Clinical features of the sample.

Psychopathological Features		
Family history of mental disorders	Yes 14 (17.5%) No 66 (82.5%)	
Length of hospitalisation (days)	Mean 20.4 (SD 8.4) Median 15.0 3–150	
Diagnosis (ICD-10)	schizophrenia spectrum disorder (F20) 52 (65%) psychotic disorders due to psychoactive substance use (F 19.5) 20 (25%) manic episode with psychotic symptoms 3, severe depressive episode with psychotic symptoms 2 (F 30.2, F 32.3) (6.3%) unspecified nonorganic psychosis) (F29) 2 (2.5%)	
Comorbidities	No comorbidities 46 (57.5 %) Pervasive developmental disorders (F84) 3 (3.75%) Antisocial personality disorder (F60.2) 1 (1.25 %) Internet use disorder 1 (F 63.8) (1.25 %) Intellectual disability 2 (F 70) (2.5 %) Obsessive-compulsive disorder (F42) 1 (1.25 %) Borderline personality disorder 1 (F 60.3) (1.25 %) Gambling disorder 1 (F63.0) (1.25 %)	
Substance use disorder	Substance use disorder 28 Alcohol 14 (F10) (50%) Cannabis 10 (F12) (35.7%) Cocaine 8 (F14) (28.5%) Alcohol and cocaine 5 (17.9%)	
Previous contact with mental health services (psychotherapies, being in charge of mental health services)	Yes 38 (47.5%) No 42 (52.5%)	
Compulsory hospitalisation	Yes 28 (35%) No 52 (65%)	
Concomitant therapies at discharge	Antidepressants 5 (6.3%) Mood stabilisers 14 (17.5%)	
Type of discharge plan	Home 61 (76.3%) Prison 2 (2.5%) Residential facility 17 (21.3%)	
Formulation of an antipsychotic treatment	LAI 35 (43.8%) Oral antipsychotic treatment 45 (56.3%)	
Types of antipsychotics used	LAI 11 Aripiprazole (31.4%) 10 Haloperidol (28.6%) 9 Paliperidone (25.7%) 4 Risperidone (11.4%) 1 Olanzapine (2.9%)	Oral 8 (17.8%) Aripiprazole 7 (15.6%) Olanzapine 7 (15.6%) Risperidone 7 (15.6%) Clozapine 5 (11.1%) Haloperidol 4 (8.9%) Paliperidone 3 (6.6%) Brexpiprazole 2 (4.4%) Cariprazine 1 (2.2%) Lurasidone 1 (2.2%) Quetiapine

**Table 3:** Readmission rates and PANSS total scores of the sample at baseline and follow-up.

Readmission	T1 (6 months)	T2 (1 year)
	19 (23.8%) Yes	36 (45%) Yes
	61 (76.2%) No	44 (55%) No
PANSS scores at discharge (mean, SD, range)	136 (11) 90–160	
Readmitted two times (N = 5)	3 M, 2 F	
	Mean age 20.4 (2.3) 18–24	
	3 Neet, 1 student, and 1 unemployed	
	4 Single, 1 married or in a relationship	
	3 No comorbidities, 2 substance use disorder	

**Table 4:** Binomial regression model (Model 1) Estimates represent the log odds of “Readmitted within 1 year = No” vs. “Readmitted within 1 year = Yes.

Predictor	Beta	SE	Wald	*p*	Odds Ratio
Substance use disorder	1.07	0.52	4.17	0.04	2.92
Therapy at discharge (LAI vs. Oral)	1.60	0.54	8.82	0.003	4.95

## Data Availability

The original contributions presented in the study are included in the article; further inquiries can be directed to the corresponding author.

## References

[1] Patel R., Wilson R., Jackson R., Ball M., Shetty H., Broadbent M., Stewart R., McGuire P., Bhattacharyya S. (2016). Association of cannabis use with hospital admission and antipsychotic treatment failure in first episode psychosis: An observational study. BMJ Open.

[2] Solmi M., Radua J., Olivola M., Croce E., Soardo L., de Pablo G.S., Shin J.I., Kirkbride J.B., Jones P., Kim J.H. (2022). Age at onset of mental disorders worldwide: Large-scale meta-analysis of 192 epidemiological studies. Mol. Psychiatry.

[3] Fusar-Poli P., Borgwardt S., Bechdolf A., Addington J., Riecher-Rössler A., Schultze-Lutter F., Keshavan M., Wood S., Ruhrmann S., Seidman L.J. (2013). The psychosis high-risk state: A comprehensive state-of-the-art review. JAMA Psychiatry.

[4] Fusar-Poli P., Tantardini M., De Simone S., Ramella-Cravaro V., Oliver D., Kingdon J., Kotlicka-Antczak M., Valmaggia L., Lee J., Millan M. (2017). Deconstructing vulnerability for psychosis: Meta-analysis of environmental risk factors for psychosis in subjects at ultra high-risk. Eur. Psychiatry.

[5] Raballo A., Poletti M., Preti A., McGorry P. (2022). Clinical high risk for psychosis in children and adolescents: A meta-analysis of transition prevalences. Schizophr. Res..

[6] Fusar-Poli P., Spencer T., De Micheli A., Curzi V., Nandha S., McGuire P. (2020). Outreach and support in South-London (OASIS) 2001–2020: Twenty years of early detection, prognosis and preventive care for young people at risk of psychosis. Eur. Neuropsychopharmacol..

[7] McGorry P.D. (2015). Early intervention in psychosis: Obvious, effective, overdue. J. Nerv. Ment. Dis..

[8] Pelizza L., Leuci E., Quattrone E., Paulillo G., Pellegrini P. (2023). The ‘Parma At-Risk mental states’ (PARMS) program: General description and process analysis after 5 years of clinical activity. Early Interv. Psychiatry.

[9] Howes O.D., Whitehurst T., Shatalina E., Townsend L., Onwordi E.C., Mak T.L.A., Arumuham A., O’brien O., Lobo M., Vano L. (2021). The clinical significance of duration of untreated psychosis: An umbrella review and random-effects meta-analysis. World Psychiatry.

[10] Sheitman B., Lieberman J. (1998). The natural history and pathophysiology of treatment resistant schizophrenia. J. Psychiatr. Res..

[11] Harrigan S.M., McGORRY P.D., Krstev H. (2003). Does treatment delay in first-episode psychosis really matter?. Psychol. Med..

[12] Ienciu M., Romoşan F., Bredicean C., Romoşan R. (2010). First episode psychosis and treatment delay—Causes and conse-quences. Psychiatr. Danub..

[13] Stirling J., White C., Lewis S., Hopkins R., Tantam D., Huddy A., Montague L. (2003). Neurocognitive function and outcome in first-episode schizophrenia: A 10-year follow-up of an epidemiological cohort. Schizophr. Res..

[14] Salem M.O., Moselhy H.F., Attia H., Yousef S. (2009). Psychogenic Psychosis Revisited: A Follow up Study. Int. J. Health Sci..

[15] Colizzi M., Cullen A.E., Martland N., Di Forti M., Murray R., Schoeler T., Bhattacharyya S. (2023). Association between stressful life events and psychosis relapse: A 2-year prospective study in first-episode psychosis. World Psychiatry.

[16] Rumball-Smith J., Hider P. (2009). The validity of readmission rate as a marker of the quality of hospital care, and a recom-mendation for its definition. New Zealand Med. J..

[17] Brown E., Bedi G., McGorry P., O’donoghue B. (2020). Rates and Predictors of Relapse in First-Episode Psychosis: An Australian Cohort Study. Schizophr. Bull. Open.

[18] Tedeschi F., Donisi V., Salazzari D., Cresswell-Smith J., Wahlbeck K., Amaddeo F. (2020). Clinical and organizational factors predicting readmission for mental health patients across Italy. Soc. Psychiatry Psychiatr. Epidemiol..

[19] Dey S., Menkes D.B., Obertova Z., Chaudhuri S., Mellsop G. (2016). Correlates of rehospitalisation in schizophrenia. Australas. Psychiatry.

[20] Edgcomb J.B., Sorter M., Lorberg B., Zima B.T. (2020). Psychiatric readmission of children and adolescents: A systematic review and meta-analysis. Psychiatr. Serv..

[21] Strålin P., Skott M., Cullberg J. (2021). Early predictors for late hospitalizations up to 14 years after first episode psychosis. Soc. Psychiatry Psychiatr. Epidemiol..

[22] Correll C.U. (2014). Recognition of patients who would benefit from LAI antipsychotic treatment: How to assess adherence. J. Clin. Psychiatry.

[23] Poloni N., Ielmini M., Caselli I., Lucca G., Gasparini A., Gasparini A., Lorenzoli G., Callegari C. (2019). Oral antipsychotic versus long-acting injections antipsychotic in schizophrenia spectrum disorder: A mirror analysis in a real-world clinical setting. Psychopharmacol. Bull..

[24] Emsley R., Chiliza B., Asmal L., Mashile M., Fusar-Poli P. (2013). Long-acting injectable antipsychotics in early psychosis: A literature review. Early Interv. Psychiatry.

[25] Medrano S., Abdel-Baki A., Stip E., Potvin S. (2018). Three-Year Naturalistic Study on Early Use of Long-Acting Injectable Antipsychotics in First Episode Psychosis. Psychopharmacol. Bull..

[26] Abdel-Baki A., Thibault D., Medrano S., Stip E., Ladouceur M., Tahir R., Potvin S. (2020). Long-acting antipsychotic medication as first-line treatment of first-episode psychosis with comorbid substance use disorder. Early Interv. Psychiatry.

[27] Farrelly S., Harris M.G., Henry L.P., Purcell R., Prosser A., Schwartz O., Jackson H., McGorry P.D. (2007). Prevalence and correlates of comorbidity 8 years after a first psychotic episode. Acta Psychiatr. Scand..

[28] Addington J., Addington D. (2007). Patterns, predictors and impact of substance use in early psychosis: A longitudinal study. Acta Psychiatr. Scand..

[29] Sorbara F., Liraud F., Assens F., Abalan F., Verdoux H. (2003). Substance use and the course of early psychosis: A 2-year follow-up of first-admitted subjects. Eur. Psychiatry.

[30] Cervone A., D’Onghia A., Ferrara M., Massaro C.R.M. (2015). Efficacy of LAI in first episode psychosis: An observational study—Clinical reports. Psychiatr. Danub..

[31] Malla A., Norman R., Bechard-Evans L., Schmitz N., Manchanda R., Cassidy C. (2008). Factors influencing relapse during a 2-year follow-up of first-episode psychosis in a specialized early intervention service. Psychol. Med..

[32] Wade D., Harrigan S., Edwards J., Burgess P.M., Whelan G., McGorry P.D. (2006). Substance misuse in first-episode psychosis: 15-month prospective follow-up study. Br. J. Psychiatry.

[33] Ceceli A.O., Bradberry C.W., Goldstein R.Z. (2022). The neurobiology of drug addiction: Cross-species insights into the dysfunction and recovery of the prefrontal cortex. Neuropsychopharmacology.

[34] Goldstein R.Z., Volkow N.D. (2011). Dysfunction of the prefrontal cortex in addiction: Neuroimaging findings and clinical implications. Nat. Rev. Neurosci..

[35] Lambert M., Conus P., Lubman D.I., Wade D., Yuen H., Moritz S., Naber D., McGorry P.D., Schimmelmann B.G. (2005). The impact of substance use disorders on clinical outcome in 643 patients with first-episode psychosis. Acta Psychiatr. Scand..

[36] Weibell M.A., Hegelstad W.T.V., Auestad B., Bramness J., Evensen J., Haahr U., Joa I., Johannessen J.O., Larsen T.K., Melle I. (2017). The Effect of Substance Use on 10-Year Outcome in First-Episode Psychosis. Schizophr. Bull..

[37] Böckmann V., Lay B., Seifritz E., Kawohl W., Roser P., Habermeyer B. (2019). Patient-Level Predictors of Psychiatric Readmission in Substance Use Disorders. Front. Psychiatry.

[38] Lin C.-H., Chen W.-L., Lin C.-M., Lee M.-D., Ko M.-C., Li C.-Y. (2010). Predictors of psychiatric readmissions in the short- and long-term: A population-based study in taiwan. Clinics.

[39] Hung Y.-Y., Chan H.-Y., Pan Y.-J. (2017). Risk factors for readmission in schizophrenia patients following involuntary admission. PLoS ONE.

[40] Bernardo A.C., Forchuk C. (2001). Factors associated with readmission to a psychiatric facility. Psychiatr. Serv..

[41] Sugisawa S., Kurihara T., Nakano Y., Tsuneoka T., Koya H., Nagai T., Ikeda T., Fujisawa N., Inamoto A., Iwanami A. (2022). Risk factors for readmission in schizophrenia treated with combined psychoeducation and standard therapy. Neuropsychopharmacol. Rep..

[42] Portela R., Wainberg M.L., Castel S., de Oliveira H.N., Ruas C.M. (2022). Risk factors associated with readmissions of patients with severe mental disorders under treatment with antipsychotics. BMC Psychiatry.

[43] Fusar-Poli P., McGorry P.D., Kane J.M. (2017). Improving outcomes of first-episode psychosis: An overview. World Psychiatry.

[44] Enderami A., Monesi F., Zarghami M. (2017). One-Year Follow-Up of Patients with a Diagnosis of First Episode Psychosis. Mater. Socio Medica.

[45] Üçok A., Polat A., Çakır S., Genç A. (2006). One year outcome in first episode schizophrenia. Eur. Arch. Psychiatry Clin. Neurosci..

[46] Kay S.R., Fiszbein A., Opler L.A. (1987). The positive and negative syndrome scale (PANSS) for schizophrenia. Schizophr. Bull..

[47] (2022). The jamovi. The Jamovi Project (Version 2.3) [Computer Software]. https://www.jamovi.org.

[48] Robinson D.G., Schooler N.R., Rosenheck R.A., Lin H., Sint K.J., Marcy P., Kane J.M. (2019). Predictors of hospitalization of individuals with first-episode psychosis: Data from a 2-year follow-up of the RAISE-ETP. Psychiatr. Serv..

[49] Colizzi M., Burnett N., Costa R., De Agostini M., Griffin J., Bhattacharyya S. (2018). Longitudinal assessment of the effect of cannabis use on hospital readmission rates in early psychosis: A 6-year follow-up in an inpatient cohort. Psychiatry Res..

[50] Schoeler T., Petros N., Di Forti M., Klamerus E., Foglia E., Murray R., Bhattacharyya S. (2017). Poor medication adherence and risk of relapse associated with continued cannabis use in patients with first-episode psychosis: A prospective analysis. Lancet Psychiatry.

[51] Gerlach J., Koret B., Gereš N., Matić K., Prskalo-Čule D., Vrbanc T.Z., Lovretić V., Skopljak K., Matoš T., Filipčić I. (2019). Clinical challenges in patients with first episode psychosis and cannabis use: Mini-review and a case study. Psychiatr. Danub..

[52] McGinty E.E., Daumit G.L. (2020). Integrating Mental Health and Addiction Treatment into General Medical Care: The Role of Policy. Psychiatr. Serv..

[53] Brousselle A., Lamothe L., Sylvain C., Foro A., Perreault M. (2010). Integrating services for patients with mental and substance use disorders: What matters?. Health Care Manag. Rev..

[54] Patel M.X., Bent-Ennakhil N., Sapin C., di Nicola S., Loze J.-Y., Nylander A.-G., Heres S. (2020). Attitudes of European physicians towards the use of long-acting injectable antipsychotics. BMC Psychiatry.

[55] Besenius C., Clark-Carter D., Nolan P. (2010). Health professionals’ attitudes to depot injection antipsychotic medication: A systematic review. J. Psychiatr. Ment. Health Nurs..

[56] Lindenmayer J.-P., Glick I.D., Talreja H., Underriner M. (2020). Persistent Barriers to the Use of Long-Acting Injectable Antipsychotics for the Treatment of Schizophrenia. J. Clin. Psychopharmacol..

[57] Kane J.M., Schooler N.R., Marcy P., Correll C.U., Achtyes E.D., Gibbons R.D., Robinson D.G. (2020). Effect of Long-Acting Injectable Antipsychotics vs Usual Care on Time to First Hospitalization in Early-Phase Schizophrenia. JAMA Psychiatry.

[58] Schreiner A., Aadamsoo K., Altamura A.C., Franco M., Gorwood P., Neznanov N.G., Schronen J., Ucok A., Zink M., Janik A. (2015). Paliperidone palmitate versus oral antipsychotics in recently diagnosed schizophrenia. Schizophr. Res..

[59] Marcus S.C., Zummo J., Pettit A.R., Stoddard J., Doshi J.A. (2015). Antipsychotic adherence and rehospitalization in schizophrenia patients receiving oral versus long-acting injectable antipsychotics following hospital discharge. J. Manag. Care Spec. Pharm..

[60] Lin C.-H., Chen F.-C., Chan H.-Y., Hsu C.-C. (2019). Time to Rehospitalization in Patients With Schizophrenia Receiving Long-Acting Injectable Antipsychotics or Oral Antipsychotics. Int. J. Neuropsychopharmacol..

[61] Vita A., Fagiolini A., Maina G., Mencacci C., Spina E., Galderisi S. (2023). Achieving long-term goals through early personalized management of schizophrenia: Expert opinion on the role of a new fast-onset long-acting injectable antipsychotic. Ann. Gen. Psychiatry.

[62] Iwata N., Inagaki A., Sano H., Niidome K., Kojima Y., Yamada S. (2020). Treatment Persistence Between Long-Acting Injectable Versus Orally Administered Aripiprazole Among Patients with Schizophrenia in a Real-World Clinical Setting in Japan. Adv. Ther..

[63] Arango C., Fagiolini A., Gorwood P., Kane J.M., Diaz-Mendoza S., Sahota N., Correll C.U. (2023). Delphi panel to obtain clinical consensus about using long-acting injectable antipsychotics to treat first-episode and early-phase schizophrenia: Treatment goals and approaches to functional recovery. BMC Psychiatry.

[64] Ishigooka J., Nakamura J., Fujii Y., Iwata N., Kishimoto T., Iyo M., Uchimura N., Nishimura R., Shimizu N. (2015). Efficacy and safety of aripiprazole once-monthly in Asian patients with schizophrenia: A multicenter, randomized, double-blind, non-inferiority study versus oral aripiprazole. Schizophr. Res..

[65] Owusu E., Oluwasina F., Nkire N., Lawal M.A., Agyapong V.I.O. (2022). Readmission of Patients to Acute Psychiatric Hospitals: Influential Factors and Interventions to Reduce Psychiatric Readmission Rates. Healthcare.

[66] Haywood T.W., Kravitz H.M., Grossman L.S., Cavanaugh J.L., Davis J.M., Lewis D.A. (1995). Predicting the “revolving door” phenomenon among patients with schizophrenic, schizoaffective, and affective disorders. Am. J. Psychiatry.

[67] Ruggeri M., Lora A., Semisa D. (2008). The SIEP-DIRECT’s Project on the discrepancy between routine practice and evidence. An outline of main findings and practical implications for the future of community based mental health services. Epidemiol. Psychiatr. Sci..

[68] Kalseth J., Lassemo E., Wahlbeck K., Haaramo P., Magnussen J. (2016). Psychiatric readmissions and their association with environmental and health system characteristics: A systematic review of the literature. BMC Psychiatry.

[69] Baeza I., Fortea A., Ilzarbe D., Sugranyes G. (2023). What Role for Long-Acting Injectable Antipsychotics in Managing Schizophrenia Spectrum Disorders in Children and Adolescents? A Systematic Review. Pediatr. Drugs.

